# A capsule network-based public health prediction system for chronic diseases: clinical and community implications

**DOI:** 10.3389/fpubh.2025.1526360

**Published:** 2025-03-14

**Authors:** Haiyan Xie

**Affiliations:** Medical College of Changsha Social Work College, Changsha, China

**Keywords:** capsule network, information system, public health, chronic disease, forecast

## Abstract

**Objective:**

To observe the role of a public health chronic disease prediction method based on capsule network and information system in clinical treatment and public health management.

**Methods:**

Patients with hypertension, diabetes, and asthma admitted from May 2022 to October 2023 were incorporated into the research. They were grouped into hypertension group (*n* = 341), diabetes group (*n* = 341), and asthma group (*n* = 341). The established chronic disease prediction method was used to diagnose these types of public health chronic diseases. The key influencing factors obtained by the prediction method were compared with the regression analysis results. In addition, its diagnostic accuracy and specificity were analyzed, and the clinical diagnostic value of this method was explored. This method was applied to public health management and the management approach was improved based on the distribution and prevalence of chronic diseases. The effectiveness and residents’ acceptance of public health management before and after improvement were compared, and the application value of this method in public health management was explored.

**Results:**

The key factors affecting the three diseases obtained by the application of prediction methods were found to be significantly correlated with disease occurrence after regression analysis (*p* < 0.05). Compared with before application, the diagnostic accuracy, specificity and sensitivity values of the method were 88.6, 89 and 92%, respectively, which were higher than the empirical diagnostic methods of doctors (*p* < 0.05). Compared with other existing AI-based chronic disease prediction methods, the AUC value of the proposed method was significantly higher than theirs (*p* < 0.05). This indicates that the diagnostic method proposed in this study has higher accuracy. After applying this method to public health management, the wellbeing of individuals with chronic conditions in the community was notably improved, and the incidence rate was notably reduced (*p* < 0.05). The acceptance level of residents toward the management work of public health management departments was also notably raised (*p* < 0.05).

**Conclusion:**

The public health chronic disease prediction method based on information systems and capsule network has high clinical value in diagnosis and can help physicians accurately diagnose patients’ conditions. In addition, this method has high application value in public health management. Management departments can adjust management strategies in a timely manner through predictive analysis results and propose targeted management measures based on the characteristics of residents in the management community.

## 1 Introduction

With the continuous development of the national economy, more and more people are willing to go to hospitals for diagnosis and treatment, and hospitals need to process a large amount of patient related information every day. This information is of great significance for predicting chronic diseases in public health (1, 2). If data mining and machine learning technologies can be utilized to further utilize the relevant diagnosis and treatment information in hospital information systems, it will further assist doctors in medical diagnosis (3–5). Chronic public health diseases include hypertension, diabetes, heart disease, etc. The occurrence of these diseases is closely related to personal lifestyle, genetic factors, environmental factors, etc. (6, 7). Accurately predicting the occurrence of these chronic diseases is of great significance for early intervention, reducing medical costs, and improving people’s quality of life. However, traditional chronic disease prediction methods mainly rely on doctors’ experience for evaluation, which is not highly accurate and time-consuming (8, 9). In addition, when formulating corresponding management measures, public health departments need to have a comprehensive understanding of the health status of residents in the current area. The current public health management work relies on manual statistical analysis by relevant personnel based on past experience data, which is inefficient and prone to bias and omission in the data statistics process (10). If artificial intelligence algorithms can be applied to further examine data in information systems, it will effectively assist the management work of public health departments, improve management efficiency, and avoid decision-making errors caused by data omissions and other situations (11). With the continuous development of science and technology, artificial intelligence has gradually been applied to the prediction of chronic diseases. In order to select A machine learning model suitable for their dataset in medical research, Huang et al. evaluated the variance of the model metrics calculated through bootstrap simulations and SHapely Additive exPlanations. By comparing the variance values, it is found that XGBoost model shows high stability and accuracy in the prediction of heart disease. Its balance accuracy ranges from 0.688 to 0.894 (12). Lee et al. explored the performance expansion direction of machine learning-based disease prediction models, established four chronic disease prediction models with commonly used data, and analyzed their scalability possibilities through analysis tools. The results show that the prediction accuracy of logistic regression, gradient enhancement, random forest and extreme gradient enhancement models exceeds 80%, especially for chronic diseases such as hypertension and diabetes (13). Huang et al. proposed a machine learning model based on XGBoost in order to predict and identify sleep disorders, and sorted the insomnia prediction results output by the model according to coverage statistics to determine the risk factors for insomnia. The results show that the area under the operator acceptance curve of this method is 0.87 (14). Based on the above research content, it can be seen that although the current AI-based chronic disease prediction method can make some progress in improving the accuracy and efficiency of prediction, the quality and integrity of data have a great impact on it. And its prediction accuracy and data processing time still have a large room for improvement. Existing machine learning methods such as XGBoost can experience performance bottlenecks when dealing with large data sets. The Capsule Network (CapsNet), through its unique dynamic routing mechanism, is able to better capture the hierarchical relationship and spatial structure in the data, which shows potential advantages when dealing with complex medical data. In response to this situation, a public health chronic disease prediction method based on Capsule Network (CapsNet) and information system is proposed to assist physicians in diagnosing patient diseases and help public health departments develop appropriate management strategies. Through experimental analysis, the impact of this method on the therapy of chronic diseases in community health and community public health governance is studied and explored. The innovation of the research lies in the utilization of artificial intelligence and big data to establish a predictive model for chronic diseases in public health, and its application to medical diagnosis and public health management. This provides new research ideas for public health management work. This study aims to propose a new method for predicting chronic diseases with higher accuracy and efficiency, and verify its effectiveness and feasibility in application, so as to provide a scientific basis for the early diagnosis of chronic diseases and public health management.

## 2 Methods

### 2.1 General information

A large general hospital in Northeast China is selected as the research object for the reason that this geographical location has a high incidence of chronic diseases and a diverse population structure, which can provide abundant research samples. In addition, general hospitals can represent the current general level of chronic disease management, and have a sufficient sample size of patients to ensure the universality and reliability of research results.

In terms of study design, the study first screened eligible patients with hypertension, diabetes and asthma through the hospital information system, and determined the final study objects according to the inclusion and exclusion criteria. These patients were divided into three study groups according to disease type, and the data were collected and analyzed, respectively. During the study, clinical data, laboratory test results and follow-up data of patients were collected to ensure the comprehensiveness and accuracy of the study results.

The research selected hypertension, diabetes and asthma patients admitted from May 2022 to October 2023 as the study subjects. The reason for choosing patients with hypertension, diabetes and asthma as study objects is that these three types of chronic diseases are extremely common in modern society and affect the living standards of sufferers (15–18). These three diseases have significant typicality as chronic diseases in public health. Therefore, studying their prevalence, treatment effectiveness, and management strategies is of great significance for improving public health.

The inclusion criteria for hypertensive patients are as follows: ① Systolic blood pressure ≥ 140 mmHg, diastolic blood pressure ≥ 90 mmHg; ② Age above 18 years old and below 80 years old; ③ Not receiving systematic treatment or non-standard treatment, resulting in poor blood pressure control. Exclusion criteria: ① Suffering from serious complications such as chronic obstructive pulmonary disease; ② Severe liver and kidney dysfunction; ③ There are mental illnesses present. The inclusion criteria of patients with diabetes are as follows: ① Between 18 and 80 years old; ② Glycated hemoglobin is 7.0%–10.0%; ③ Capable of comprehending and executing consent documents that detail the information provided. Exclusion criteria: ① Suffering from severe heart, liver, and kidney dysfunction; ② There are acute complications of diabetes, such as ketoacidosis or hyperosmolar coma. The inclusion criteria for asthma patients are as follows: ① Age between 18 and 80 years old; ② Symptoms such as recurrent wheezing, shortness of breath, chest tightness, or cough, accompanied by variable expiratory flow rate limitation; ③ Not receiving systematic treatment or non-standard treatment, resulting in poor symptom control. Exclusion criteria: ① Suffering from serious complications such as chronic obstructive pulmonary disease; ② Severe liver and kidney dysfunction; ③ There are mental illnesses present. The study has been reviewed and approved by the hospital ethics committee. Table 1 presents the basic details of the study participants.

**Table 1:** Details of the study participants.

Project		Hypertension group (*n* = 341)	Diabetes group (*n* = 341)	Asthma group (*n* = 341)	*p* ^a^	*p* ^b^	*p* ^c^
Age (years)		51.32 ± 11.05	51.42 ± 10.85	51.30 ± 11.00	>0.05	>0.05	>0.05
Sex	Male	172	174	170	>0.05	>0.05	>0.05
	Female	169	167	171	>0.05	>0.05	>0.05
Body mass index (kg·m^−2^)		25.83 ± 2.44	27.22 ± 4.12	22.45 ± 2.14	<0.05	<0.05	<0.05
Duration of disease (years)		2.54 ± 1.58	2.50 ± 1.26	2.53 ± 1.34	>0.05	>0.05	>0.05

a Represents the significance of data difference between hypertension group and diabetes group.

b Represents the significance of data difference between diabetes group and asthma group.

c Represents the significance of data difference between hypertension group and asthma group.

In addition, the study also collected data on the public health situation in the region from the hospital information system from May 2022 to September 2024. By collecting relevant data of patients admitted during this period, it provided a foundation for establishing predictive models and analyzing public health management in the future. During the study, all patients received routine treatment and management, while individualized treatment plans were adjusted according to their respective conditions. Regular monitoring of patients’ blood pressure, blood glucose, and asthma symptoms was conducted, and relevant data were recorded for subsequent statistical analysis. During the treatment process, the public health chronic disease prediction model based on CapsNet and information system proposed in the research was used to adjust the treatment plan of patients in a timely manner, and the clinical treatment effects of patients before and after applying the prediction model were compared.

### 2.2 Research methods

**Figure 1:**
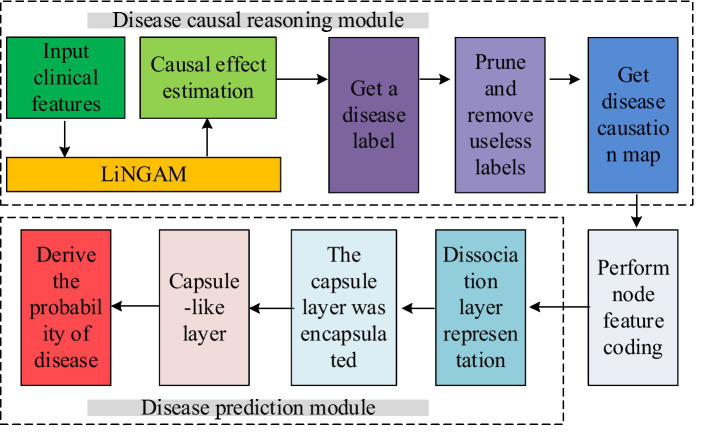
Public health chronic disease prediction method based on CapsNet and information system.

During the experiment, the study used the constructed public health chronic disease prediction method to statistically analyze the relevant data of sufferers with hypertension, diabetes and asthma in the hospital, and explore the influencing factors of these diseases. Meanwhile, by inferring the causality between clinical characteristics of diseases, the method predicted whether patients had related diseases, compared the accuracy of prediction and diagnosis results, and explored the clinical application value of this chronic disease prediction method. In addition, the study also applied it to predict the occurrence of chronic diseases in the population within the region, exploring its auxiliary role in providing management decisions for governmental health agencies. The overall framework of the public health chronic disease prediction method based on CapsNet and information system proposed in the study is shown in Figure 1.

As shown in Figure 1, the public health chronic disease prediction method proposed in the study is divided into two parts. The first part is the medical causal inference part, which constructs a causal inference model for chronic disease clinical data using a non-temporal nonlinear non-Gaussian model (LiNGAM). The second part is a disease prediction model based on CapsNet. This model combines the results of the disease causality diagram constructed in the first part to correlate diseases with relevant clinical features, and then predict the probability of disease occurrence. In the part of causal reasoning, LiNGAM is used to calculate causal effects and causal links between clinical data, clinical data and disease labels. Then, according to the causal effects and causal links obtained, a causal graph of clinical data is constructed, and nodes in the graph that have no causal association with disease labels are deleted. Finally, the deleted disease label node is used as the class label of the graph, and then the disease causality graph is constructed. The part of disease prediction model is to realize disease prediction by binary classification of clinical data of each disease. In this study, the capsule network model was used to classify the disease causality map. In addition, a dynamic routing algorithm is introduced to improve the accuracy of feature representation and the generalization ability of the model. The dynamic routing layer uses the graph convolutional neural network to directly transform the voting process of the route into the node classification process of the node capsule, so as to realize the efficient learning of the complex relationship between clinical features and diseases. To calculate the causality between disease labels and clinical features, the study normalized the disease labels and inputted them into the LiNGAM model for causal calculation. In the causal calculation process, the residual vector between two feature data directly affects the causal ranking. Therefore, it is necessary to first calculate the residual vector and apply this result to the calculation of the independence score. The variable with the strongest independence is selected to the causal list and it will be sorted until all features have completed causal inference sorting. After obtaining the list, the study used covariance matrix regression to analyze its causal strength, obtained the strength matrix, and determined whether there was a causal relationship. The calculation method of residual vector is shown in equation 1.


(1) $$r_{i}^{j}=x_{i}-\frac{\mathrm{cov}\left(x_{i}x_{j}\right)}{\mathrm{cov}\left(x_{j}\right)}x_{i}$$

**Figure 2:**
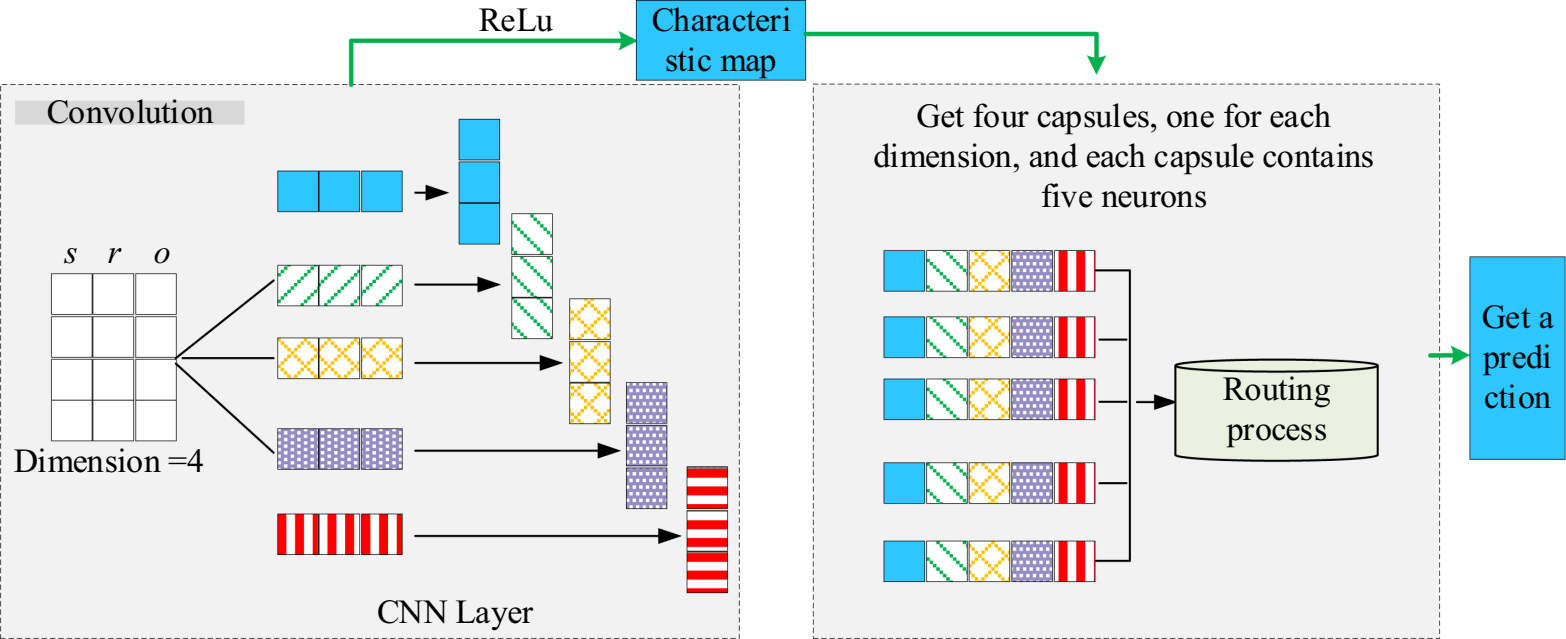
The specific structure of CapsNet.

In equation 1, var() and cov() represent the variance and covariance between variables, *x_i_* and *x_j_* represent two different sample features, and $r_{i}^{j}$ represents the residual vector during regression analysis of two sample features. Afterwards, based on the obtained causal strength matrix, the sample data is converted into a causal graph for subsequent prediction models to classify and identify the causal graph. In the disease prediction section, CapsNet was used for disease prediction, and the clinical data causal diagram constructed by the medical causal inference module was used to classify the causal diagram during the prediction process. Figure 2 illustrates the detailed architecture of CapsNet.

As shown in Figure 2, the CapsNet further extracts the spatial relationships of data features through spatial decoding operations. The study introduced graph neural networks based on the CapsNet structure to predict chronic diseases. The proposed prediction model mainly includes a dissociation representation layer, a hierarchical capsule layer, and a dynamic routing layer. In the dissociation representation layer, multiple linear transformations are applied to the node representation to extract the heterogeneous factors behind it. The hierarchical capsule layer further organizes these heterogeneous factors into capsules with a hierarchical structure, where each capsule is responsible for capturing feature information of different scales and levels. The dynamic routing layer optimizes the activation state of capsules and enhances the model’s ability to recognize disease features by iteratively transmitting information between capsules. The study applied the model to the diagnosis of chronic diseases in hospitals for a period of 6 months.

After applying chronic disease prediction methods to understand the situation of chronic diseases in the city, the public health management department formulates corresponding prevention strategies and intervention measures based on the characteristics of chronic disease distribution. Firstly, they strengthen the screening of high-risk populations, improve residents’ awareness of early symptoms of chronic diseases through regular physical examinations and health education. Secondly, the public health management department collaborates with local medical institutions to optimize the treatment plans for chronic disease patients, ensuring that they can receive sustained and effective medical services. In addition, they promote a healthy lifestyle in community management, encourage residents to participate in physical exercise, improve dietary habits, and reduce the risk factors of chronic diseases such as obesity and hypertension. During the implementation of measures, public health management departments use chronic disease prediction methods to track the incidence and treatment effectiveness of chronic diseases in real time. Based on the analysis results, strategies are adjusted in a timely manner to ensure the most effective utilization of resources.

### 2.3 Observation indicators

Twenty two blood routine indicators and 15 blood biochemical indicators: including white blood cell count, red blood cell count, hemoglobin concentration, platelet count, neutrophil ratio, lymphocyte ratio, eosinophil ratio, eosinophil ratio, red blood cell distribution width, average red blood cell volume, serum sodium, serum potassium, serum chloride, serum calcium, etc.

**Regression analysis results:** The key indicators that affect the occurrence of these chronic diseases are explored and the rationality of the key indicators derived from causal relationships in the predictive model proposed in the study is tested.

**Diagnostic accuracy:** the most basic indicator for evaluating the quality of prediction methods, referring to the degree of conformity between predicted results and actual occurrences. High accuracy means that prediction methods can accurately identify which patients will develop chronic diseases in the future.

**Sensitivity and specificity:** Sensitivity denotes the fraction of patients with actual illness that can be correctly identified by predictive methods. High sensitivity means less-missed diagnosis rate. Specificity refers to the proportion of patients who are not actually ill and can be correctly identified by the prediction method. High specificity means less misdiagnosis rate.

**Positive Predictive Value (PPV):** A PPV denotes the proportion of actual illness among all predicted positive outcomes. A high positive predictive value means that when the prediction result is positive, the likelihood of the patient actually getting sick is high.

**Negative Predictive Value (NPV):** An NPV refers to the proportion of results that are forecasted as negative and are in fact not affected by the disease. A high negative predictive value means that when the prediction result is negative, there is a high likelihood that the patient is truly not ill.

**Incidence rate:** the number of people with a certain type of chronic disease in the community calculated as a ratio of the entire quantity of people in the community.

**Patient quality of life assessment:** Physicians score the quality of life of patients through questionnaire surveys, daily activity records, and medical examination results. The score range is [0, 100], with higher scores suggesting an improved standard of living for patients.

**Resident satisfaction:** The proportion value obtained by dividing the number of residents satisfied with the management work by the total number of residents through voting.

**F1-mearsure:** Comprehensive consideration of sensitivity and specificity, is a weighted average index of sensitivity and specificity. The higher the F1-measure value, the better the overall performance of the prediction model in recognizing chronic diseases.

**Matthews Correlation Coefficient (MCC):** A measure of the performance of a prediction model that considers true cases, false positive cases, true negative cases, and false negative cases, with MCC values ranging from −1 to +1, where +1 represents a perfect prediction, 0 represents a random prediction, and −1 represents the exact opposite prediction.

The data during the research process were analyzed using SPSS 26.0, and descriptive statistical analysis methods were utilized to organize and summarize the fundamental attributes of the data. By calculating statistical indicators such as mean, standard deviation, frequency, and percentage, the distribution of the sample can be understood.

## 3 Results

### 3.1 Correlation analysis between clinical blood indicators and chronic diseases

**Figure 3:**
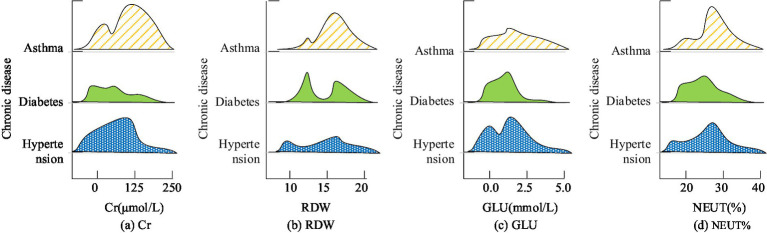
Correlation analysis between clinical blood indexes and chronic diseases. The higher the height of the graph indicates the size of the crowd under this index value, and the higher the number indicates the larger the number. **(a)** Cr; **(b)** RDW; **(c)** GLU; **(d)** NEUT%.

The study selected the top four indicators related to chronic diseases, including creatinine (Cr), red cell distribution width (RDW), glucose (GLU), and neutrophil percentage (NEUT%), and conducted statistical analysis on the detection results of these indicators in different patients. The outcome is represented in Figure 3. From Figure 3, the distribution range of clinical indicators for different chronic diseases is different, indicating that it is feasible to apply clinical blood indicators to disease prediction in this study.

### 3.2 Analysis of related clinical characteristics and prediction model rationality affecting diabetes

Seven indicators with the highest correlation with diabetes were obtained by screening causal reasoning in the prediction model. The study used Spearman correlation analysis to conduct a single factor analysis, further supporting the rationality of the proposed method in conducting causal relationships. The specific outcomes are shown in Table 2. The incidence of diabetes was positively associated with gender, serum total cholesterol, triacylglycerol and uric acid content, and negatively correlated with total bile acid and high-density lipoprotein cholesterol, with a difference of statistical significance (*p* < 0.05). There was no significant difference between body mass index (BMI) and the incidence of diabetes (*p* > 0.05).

**Table 2:** Outcomes of single factor analysis affecting the occurrence of diabetes mellitus.

Variable	*r*	*p*
Sex	0.198	0.012
BMI	0.152	0.052
Serum total cholesterol	0.154	0.046
Triacylglycerol	0.238	0.001
High density lipoprotein cholesterol	−0.178	0.028
Uric acid	0.288	0.001
Total bile acids	−0.263	0.005

The results of multi-factor correlation analysis affecting the incidence of diabetes are shown in Table 3. The study used indicators such as gender and BMI as independent variables, and whether the patient developed the disease as the dependent variable. Binary logistic regression analysis was utilized to assess the influencing factors. From Table 3, total bile acids reduced the risk of disease in patients by 49.1%, while serum total cholesterol and uric acid increased the risk of disease (*p* < 0.05).

**Table 3:** Outcomes of correlation analysis of multiple factors affecting the incidence of diabetes.

Variable	Odds ratio	95%Confidence interval	*p*
Sex	0.500	0.208–1.209	0.127
BMI	1.072	0.957–1.210	0.252
Serum total cholesterol	1.926	1.066–3.547	0.031
Triacylglycerol	1.008	1.000–1.008	0.037
Uric acid	0.506	0.285–0.912	0.023
Total bile acids	1.142	0.801–1.633	0.458

### 3.3 Analysis of relevant clinical characteristics and prediction model rationality influencing hypertension symptoms

Similarly, the study used causal inference in predictive models to screen the eight indicators with the highest association with hypertension, and conducted univariate analysis using Spearman correlation analysis, further supporting the rationality of the proposed method in conducting causal relationships. The specific outcomes are represented in Table 4. From Table 4, gender, age, alcohol consumption history, smoking history, BMI, high-density lipoprotein cholesterol, low-density lipoprotein cholesterol, and high total cholesterol were all risk factors related to hypertension.

**Table 4:** Results of single factor analysis affecting the occurrence of hypertension.

Variable	*r*	*p*
Sex	0.211	<0.05
BMI	0.357	<0.01
Age	0.182	<0.05
Drinking history	0.234	<0.05
Smoking history	0.420	<0.01
Low density lipoprotein cholesterol	−0.192	<0.05
High density lipoprotein cholesterol	0.385	<0.01
Total cholesterol	0.452	<0.01

The results of the logistic multivariate analysis on chronic public health diseases such as hypertension are shown in Table 5. From Table 5, a total of eight factors, including gender, age, and alcohol consumption history, were independent risk factors for hypertension. Age, BMI, low-density lipoprotein cholesterol, and total cholesterol were all positively correlated with hypertension, while high-density lipoprotein cholesterol was negatively correlated with hypertension complications.

**Table 5:** Analysis results of multiple factors affecting the occurrence of hypertension.

Variable	Odds ratio	95%Confidence interval	Walds	Standard error	*p*
Sex	1.342	1.022–1.875	9.322	0.230	<0.05
BMI	3.921	1.890–5.472	49.833	0.625	<0.05
Age	1.690	1.295–2.788	14.235	0.281	<0.05
Drinking history	3.901	1.421–3.876	32.25	0.415	<0.05
Smoking history	2.353	1.468–4.372	37.46	0.425	<0.05
Low density lipoprotein cholesterol	1.577	1.170–1.923	17.392	0.267	<0.05
High density lipoprotein cholesterol	1.580	1.330–3.058	12.558	0.314	<0.05
Total cholesterol	1.975	1.385–3.372	21.432	0.368	<0.05

### 3.4 Analysis of relevant clinical characteristics and prediction model rationality that affect asthma

**Figure 4:**
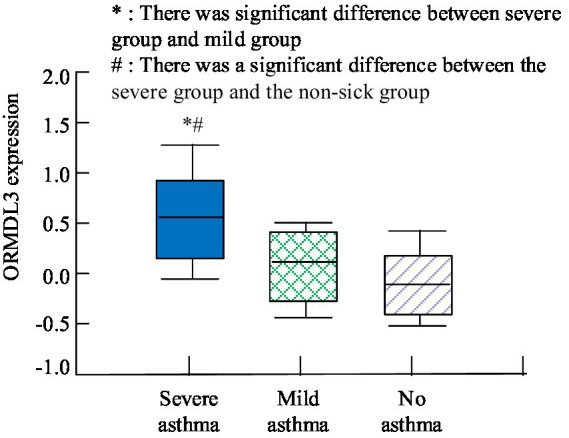
Comparison of ORMDL3 expression in different asthma conditions.

The study used causal inference in predictive models to screen the indicators with the highest association with asthma disease, including plasma Oromucoid 1-like 3 (ORMDL3), which has a significant correlation with asthma disease in terms of gene expression. To demonstrate the rationality of the results obtained from the causal screening method proposed in the study, the asthma patients included in the study were subdivided grounded on the frequency of wheezing in the past year. If there have been more than or equal to 4 instances of wheezing in the past year, it is classified as severe asthma; if there have been 1–3 instances of wheezing in the past year, it is classified as mild asthma. The differential expression of ORMDL3 was compared with patients without asthma disease. The outcome is shown in Figure 4. From Figure 4, the expression level of ORMDL3 was highest in patients with severe asthma, and there was a significant difference in expression level in comparison with the other two patients (*p* < 0.05).

To further analyze the clinical significance of the key influencing factors inferred by the causal reasoning method proposed in the diagnosis of asthma, a correlation analysis was conducted between their expression levels and other asthma related variables. The correlation analysis outcomes are shown in Table 6. From Table 6, the expression level of ORMDL3 was positively correlated with the patient’s personal history of eczema, wheezing, and immunoglobulin E (IgE) factors (*p* < 0.05). There was no correlation between it and gender, age, rhinitis (*p* > 0.05). It can be inferred that the ORMDL3 gene may occupied a key position in the pathogenesis of asthma, especially in allergic asthma associated with eczema and IgE levels.

**Table 6:** Analysis results of multiple factors affecting the occurrence of hypertension.

Variable	Odds ratio	95%Confidence interval	Walds	Standard error	*p*
Sex	1.342	1.022–1.875	9.322	0.230	<0.05
BMI	3.921	1.890–5.472	49.833	0.625	<0.05
Age	1.690	1.295–2.788	14.235	0.281	<0.05
Drinking history	3.901	1.421–3.876	32.25	0.415	<0.05
Smoking history	2.353	1.468–4.372	37.46	0.425	<0.05
Low density lipoprotein cholesterol	1.577	1.170–1.923	17.392	0.267	<0.05
High density lipoprotein cholesterol	1.580	1.330–3.058	12.558	0.314	<0.05
Total cholesterol	1.975	1.385–3.372	21.432	0.368	<0.05

### 3.5 Analysis of clinical diagnostic value after applying chronic disease prediction methods

**Figure 5:**
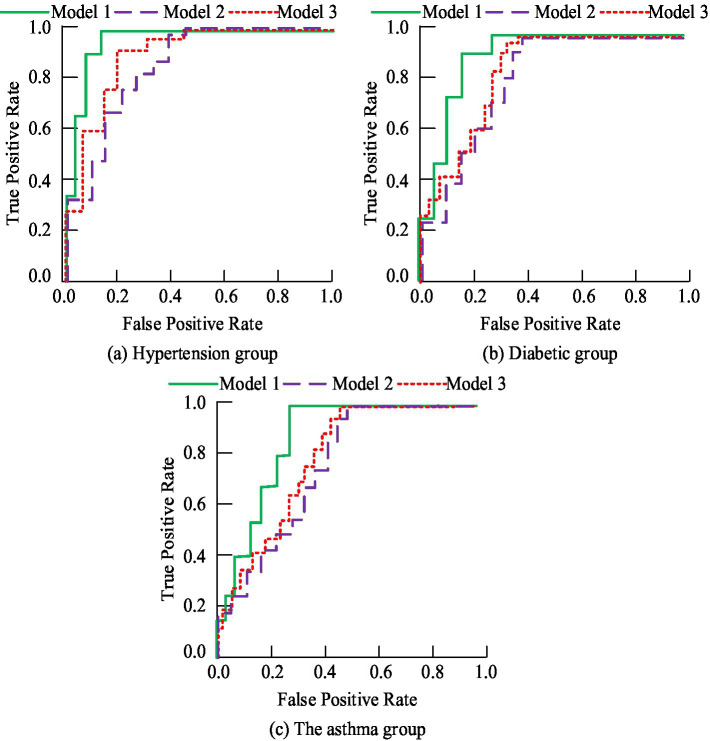
ROC curves of three models under three chronic diseases. **(a)** Hypertension group; **(b)** Diabetic group; **(c)** The asthma group.

In order to further analyze the application value of the public health chronic disease prediction model in clinical diagnosis. In the case of three diseases, the prediction model proposed in the study (Model 1) was compared with the more advanced chronic disease prediction model based on XGBoost (Model 2) and the chronic disease prediction model based on random forest (model 3). The Receiver operating curve (ROC) of the three models under the three disease conditions is shown in Figure 5. As can be seen from Figure 5, the Area under the receiver’s operating curve (AUC) of model 1 for the three chronic public health diseases all reaches above 0.9, while the average AUC of model 2 and Model 3 is 0.85 and 0.87, respectively. This shows that the prediction accuracy of the model is relatively high, which can play an auxiliary role in the diagnosis process and improve the diagnosis accuracy.

The prediction model was applied to the diagnosis of chronic diseases in hospitals, and the diagnostic accuracy, PPV, NPV, F1-measure and MCC were recorded before and after the application, as shown in Table 7. From Table 7, the initial diagnostic accuracy of doctors significantly increased before and after application, and both PPV and NPV values were higher than before application. The F1-measure value and MCC index value after the application of the diagnosis method were significantly higher than those before the application of the prediction diagnosis (*p* < 0.05). This shows that the chronic disease prediction method proposed in this study can effectively improve the diagnostic ability of doctors and reduce the situation of misdiagnosis and missed diagnosis. In addition, the model shows good stability and reliability in practical application, and provides a powerful auxiliary decision-making tool for doctors.

**Table 7:** Comparison of clinical diagnosis effect before and after applying chronic disease prediction method.

Project	Diagnostic accuracy (%)	PPV	NPV	F1-measure	MCC
Before application	75.3	72.1	80.5	0.78	0.52
Post application	88.6	85.4	92.3	0.89	0.76
*p*	<0.01	<0.01	<0.01	<0.01	<0.01

To further explore the application value of this prediction method in clinical diagnosis, the study compared it with the method of diagnosis based on doctor’s experience before application, and compared the sensitivity and specificity of the two methods. The outcomes are represented in Table 8. From Table 8, the specificity and sensitivity indicators of traditional empirical diagnostic methods were lower than those of the prediction method proposed in this study. Specifically, the specificity of empirical diagnosis was 75% and the sensitivity was 68%, while the specificity of this research method reached 89% and the sensitivity was as high as 92%.

**Table 8:** The specificity and sensitivity analysis results of routine empirical diagnosis and this diagnostic method.

Project	Hypertension group		Diabetic group		Asthma group	
	Specificity	Sensibility	Specificity	Sensibility	Specificity	Sensibility
This diagnostic method	89%	93%	90%	94%	88%	90%
Empirical diagnostic method	72%	66%	78%	69%	75%	69%
*p*	<0.05	<0.05	<0.05	<0.05	<0.05	<0.05

### 3.6 Analysis of the application value of prediction methods in public health management

**Figure 6:**
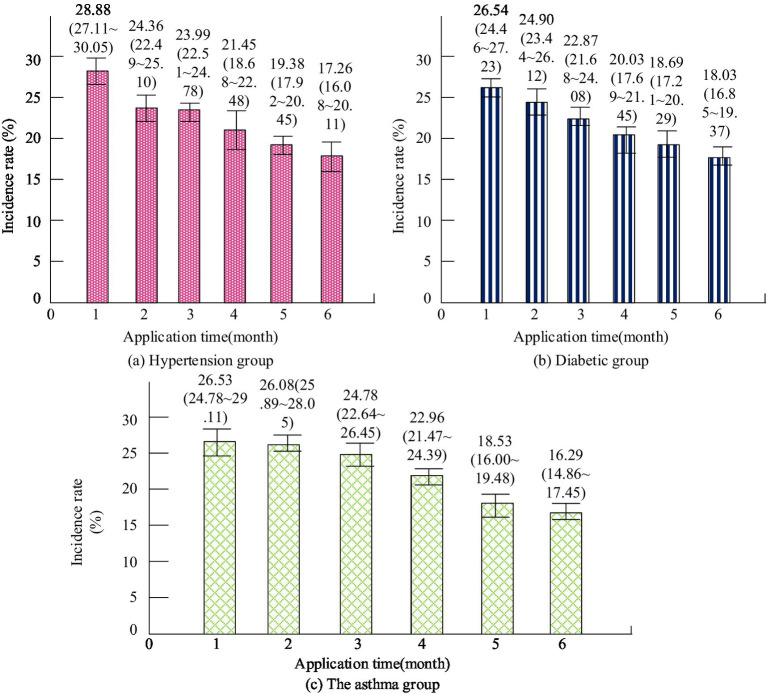
Changes in the incidence of three chronic diseases after the application of improved public health management methods.

The study applied this prediction method to public health management by inputting physical examination data and other relevant survey data of residents in the community into the prediction model, obtaining information on the prevalence and distribution of chronic diseases among residents. The management department has optimized and improved the public health management work based on the analysis results. It was applied to management work for 6 months and the changes in the incidence of diseases among community residents during this period were recorded. The change of incidence rate of three chronic diseases after the application of the improved public health management method is represented in Figure 6. From Figure 6, with the rise of application time, the incidence rate of chronic diseases of residents has been significantly reduced.

The satisfaction of community residents with management work and the improvement of quality of life for chronic diseases before and after the application of improved public health management methods are shown in Table 9. From Table 9, after improving and optimizing the management method using the prediction model, residents’ satisfaction with the management work has significantly increased (*p* < 0.05).

**Table 9:** Comparison of community residents’ satisfaction and quality of life before and after applying improved public health management methods.

Project	Hypertension group		Diabetic group		The asthma group	
	Satisfaction	Quality of life score	Satisfaction	Quality of life score	Satisfaction	Quality of life score
Before management methods are improved	0.87 ± 0.03	85.45 ± 2.41	0.86 ± 0.04	84.99 ± 2.36	0.85 ± 0.05	84.87 ± 2.38
After the improvement of management methods	0.92 ± 0.03	91.79 ± 3.22	0.93 ± 0.02	92.01 ± 3.25	0.91 ± 0.04	91.63 ± 3.48
*p*	<0.01	<0.01	<0.01	<0.01	<0.01	<0.01

In order to further verify the applicability of the proposed model to public health management, this study quantifies the resource cost savings achieved by the predictive model in public health management. The resource consumption data pairs of 6 months before and after the public health management work before and after the application of the model are shown in Table 10. As shown in Table 10, the implementation of the improved management approach resulted in significant savings in resource costs for public health management, including a 33.3 per cent reduction in human resource costs, a 33.3 per cent reduction in material costs and a 31.6 per cent reduction in management time/tasks. The statistical test showed that these differences were significant (*p* < 0.05). This indicates that the efficiency of public health management has been significantly improved by introducing the prediction model based on CapsNet, with more rational utilization of resources and obvious cost saving effect.

**Table 10:** Comparison of resource effects before and after applying the prediction model to public health management.

Project	Human resource cost (Yuan)	Cost of materials (Yuan)	Manage time/tasks (days)
Before management methods are improved	120,000	30,000	98
After the improvement of management methods	80,000	20,000	67
*p*	<0.05	<0.05	<0.05

## 4 Discussion

With the development of social economy, people’s demand for public health is also increasing. In addition to sudden outbreaks and other public health events, the mitigation and management of chronic diseases have also become a crucial element of community health management (19–21). Therefore, a public health chronic disease prediction method based on information systems and CapsNet has been proposed and applied to chronic disease diagnosis and public health management. D’Souza et al. conducted bioinformatics analysis to explore the relationship between protein interaction and diabetes. The analysis results showed that this effect would lead to an increase in the incidence of diabetes, accompanied by a decrease in high-density lipoprotein cholesterol, an increase in low-density lipoprotein cholesterol, and an increase in high total cholesterol (22). The key factors influencing diabetes analyzed in the research results included high-density lipoprotein cholesterol and low-density lipoprotein cholesterol, which was basically in alignment with the findings of the study in the above literature. In order to investigate the correlation between metal contact and the risk of diabetes, Wang et al. analyzed the potential relationship through clinical comparative experiments. The analysis results showed that the key factors affecting diabetes included gender, age, BMI and the levels of cobalt, zinc, copper, arsenic, molybdenum, cadmium, tellurium, thallium and lead (23). The key factors influencing diabetes obtained by the prediction method constructed by the research included gender and age, which was basically in alignment with the research outcomes in the above literature. Chen et al. conducted regression analysis on clinical data of middle school students in Suzhou Industrial Park to explore the key factors affecting the prevalence of hypertension among residents. The results showed that the patient’s weight, BMI index, and blood pressure were positively correlated, obesity increasing the likelihood of hypertension in students (24). When using the method proposed in the study to analyze the key factors of hypertension, it was found that BMI had a high correlation with hypertension and was positively correlated, which was in alignment with the outcomes of the literature study mentioned above. Xu et al. analyzed health survey data from 1999 to 2010 to determine the correlation between heart disease and hypertension. The analysis outcomes revealed that the cholesterol content in the blood and the ratio of neutrophils to lymphocytes were directly related to the incidence of hypertension (25). The study analyzed through predictive methods that the key factors affecting hypertension included high-density lipoprotein, low-density lipoprotein, and high total cholesterol, which were in alignment with the outcomes of the literature research mentioned above. When using the method proposed in the study to analyze the factors related to the onset of asthma, it was found that the indicator closely linked to this disease was the expression level of ORMDL3. The expression level was significantly higher in severe asthma patients (*p* < 0.05). Liu et al. investigated the main factors affecting asthma and elucidated the pathogenesis of asthma through a mouse model. Through experiments, it was found that mice with high expression levels of ORMCL3 exhibited more pronounced asthma symptoms (26). This was basically consistent with the research results.

With the development of information technology and deep learning methods, their applications in the medical field are becoming increasingly widespread (27, 28). Dileep et al. proposed a clustering-based bidirectional long short-term memory (LSTM) network algorithm to improve the accuracy of heart disease prediction. This algorithm utilized bidirectional LSTM networks for deep learning of processed data to capture time series features in heart disease data. The experiment outcomes showed that the algorithm achieved better performance than traditional prediction models on multiple heart disease datasets, with significant improvements in accuracy and recall (29). Kumar et al. proposed an autonomous edge cloud-assisted heart disease prediction framework that combined random forest and local rule generation techniques, aiming to enhance precision and effectiveness of heart disease prediction. Through testing on multiple heart disease datasets, the framework demonstrated excellent performance (30). Therefore, the study considered applying deep learning-based methods for public health chronic diseases to clinical diagnosis. After the application of the chronic disease prediction method proposed in the study in clinical diagnosis, the AUC values reached 0.9 or above, and compared with traditional doctor experience diagnosis, its diagnostic accuracy, PPV index, and NPV index were significantly higher (*p* < 0.05). In addition, the specificity and sensitivity indicators of this method were significantly higher than those of conventional methods (*p* < 0.05). This advantage is due to CapsNet’s unique capsule network structure, which better simulates how the human visual system works, resulting in superior performance when processing complex data. Its dynamic routing algorithm enables the network to learn hierarchical feature representations of the data, which is critical for understanding complex patterns in clinical data. CapsNet is able to effectively capture these hierarchical features through dynamic connections between capsules, thereby improving the accuracy of predictions. Therefore, an in-depth study of the application of CapsNet to clinical data will not only advance the development of chronic disease prediction methods, but may also provide new perspectives and tools for data analysis in other areas of medicine.

In the field of public health, the prevention and control of chronic diseases not only require effective prediction methods, but also comprehensive management strategies (31–33). In order to better address the challenges posed by chronic diseases, this method has been applied to public health management. Based on the analysis results of the predictive model, targeted optimization and improvement of public health management for residents in specific regions have been carried out. Olatunji et al. proposed a revolutionary method using rapid diagnostic technology and portable devices for managing infectious diseases in resource scarce environments. Through research, it was found that these technologies could not only improve the speed and accuracy of disease detection, but also significantly reduce disease management costs in low resource environments (34). Ransikarbum et al. proposed a central hospital location method based on clustering algorithm and vehicle routing in order to improve the management effect of infectious disease health management. The method combined with the disease data in the region to predict the disease situation, and combined with the vehicle route to select the best location of the simple medical point. The results show that the prediction accuracy of this method reaches 88% (35). It can be seen that although the existing public health management methods based on artificial intelligence can improve the management efficiency to a certain extent, they have high requirements on data quality, and the accuracy of data analysis needs to be improved. The prediction method proposed in the study could provide more reliable data judgment support. After application, community residents’ acceptance of management work was significantly improved, and the incidence rate of chronic diseases among community residents was significantly reduced (*p* < 0.05).

## 5 Strengths and limitations of the study

Based on the above content, it can be concluded that the public health chronic disease prediction method based on information systems and CapsNet plays a very positive role in clinical diagnosis and public health management, effectively helping physicians and management departments to control and prevent chronic diseases. The current study only designed the prediction method for public health chronic diseases, which has certain limitations. And the study’s reliance on hospital-specific data may limit its generality. The cross-sectional nature of the data set is a limitation of causal reasoning.

## 6 Implications of the study and future research/interventions

The significance of this study is that it provides a new perspective and tool for the prediction of chronic diseases in public health, which provides clinicians with more accurate diagnostic AIDS to help early detection and intervention of chronic diseases, thereby improving the prognosis and quality of life of patients. At the same time, the application of this method in public health management can help relevant departments to conduct health risk assessment and resource allocation more effectively, and reduce the morbidity and mortality of chronic diseases.

The implication of future studies and interventions is that as algorithms continue to be optimized and datasets expand, predictive models will be able to cover a wider range of populations and disease types. This will help build a more comprehensive public health early warning system to achieve early identification and intervention of a variety of diseases.

## 7 Recommendations

For future applications of CapsNet, the algorithm needs to be further optimized to reduce the dependence on specific data sets and improve the generalization ability of the model. In addition, the extended validation of longitudinal data sets should be considered to further explore the applicability and accuracy of the model in different populations and different settings. In order to enhance the adaptability of the model, more kinds of clinical data, such as genetic information and lifestyle data, can be introduced to enrich the input characteristics of the model. With these improvements, forecasting methods will be able to better adapt to the health needs of different regions and populations, providing stronger support for public health decision-making.

## Data Availability

The original contributions presented in the study are included in the article/supplementary material, further inquiries can be directed to the corresponding author.
